# Near extinct *Argyreia versicolor* and rare *Argyreia mekongensis* are dependent on carpenter bee pollinators

**DOI:** 10.1093/aobpla/plae001

**Published:** 2024-01-17

**Authors:** Awapa Jirabanjongjit, Paweena Traiperm, Chakkrapong Rattanamanee, Alyssa B Stewart

**Affiliations:** M.Sc. Program in Plant Sciences, Faculty of Graduate Studies, Mahidol University, Nakhon Pathom 73170, Thailand; Department of Plant Science, Faculty of Science, Mahidol University, Bangkok 10400, Thailand; Department of Pharmaceutical Botany, Faculty of Pharmacy, Mahidol University, Bangkok 10400, Thailand; Department of Plant Science, Faculty of Science, Mahidol University, Bangkok 10400, Thailand; 37/1 Moo4 Saocha-Ngok, Bangkhla, Chachoengsao 24110, Thailand; Department of Plant Science, Faculty of Science, Mahidol University, Bangkok 10400, Thailand

**Keywords:** Convolvulaceae, mating system, morning glory, pollination, self-incompatibility, *Xylocopa*

## Abstract

*Argyreia versicolor* and *Argyreia mekongensis* are extremely rare plant species. The former had not been seen for nearly 100 years until two individuals were found in Thailand in 2018, and only a handful of populations are known for the latter. The aims of this study were to examine the breeding systems of *A. versicolor* and *A. mekongensis* using pollination experiments and to determine their potential pollinators via floral observations. Our controlled pollination experiments uncovered the self-incompatibility of both species. Pollinator censuses indicated that females of two carpenter bee species, *Xylocopa aestuans* and *Xylocopa latipes*, were the predominant floral visitors for both *Argyreia* species. Our observations confirmed a harmonious match between the floral shape of both *Argyreia* species and the body sizes of these pollinators, ensuring effective pollen transfer and validating their role as putative pollinators. In line with the high frequency of pollinator visits observed, our controlled pollination experiments found no evidence of pollen limitation under field conditions. The findings of this study hold significance for the conservation of these endangered species, yet the situation is dire for *A. versicolor*, with one of the two individuals under study recently lost. Hence, it is crucial to intensify monitoring efforts for the species, aiming to identify additional individuals for potential inclusion in an ex-situ conservation program. Simultaneously, safeguarding the habitat of these plant species and their pollinators will be critical.

## Teaser

*Argyreia versicolor* and *Argyreia mekongensis* , two species of morning glory endemic to Thailand and neighbouring countries, are extremely rare and knowledge of their reproductive ecology is essential for conservation efforts. Our research found that both species are self-incompatible and require pollinators to reproduce. We also found that both morning glory species are pollinated by carpenter bees (*Xylocopa aestuans* and *Xylocopa latipes* ). Given the extreme rarity of these species (particularly *A. versicolor* , for which we know of only a single surviving individual), immediate action is needed. Successful conservation will require identifying additional individuals and safeguarding the habitat of these plant species and their pollinators.

## Introduction

Rare species are often prioritized for conservation due to their risk of extinction. An important component of plant conservation is knowing their breeding system and pollinators, as these factors directly affect reproduction (Bond 1994; Wilcock and Neiland 2002; Gargano *et al.* 2009). For example, self-incompatible species typically depend more on pollinators to set fruit than do their self-compatible counterparts (Schoen and Lloyd 1992). Moreover, self-incompatible species that are rare or occur in small populations are at greater risk of reduced reproduction due to the lack of available mates and/or insufficient pollinator visitation (Levin 1972; Kunin 1992, 1993; Delnevo *et al.* 2019). These species can also experience reduced seed set due to competition with co-occurring plant species for pollinators (Mosquin 1971; Kwak and Jennersten 1991) and/or interspecific pollen transfer due to low pollinator fidelity (Waser 1978; Thomson *et al.* 1981; Rathcke 1983; Campbell and Motten 1985). Even rare species that are self-compatible may be negatively affected, such as incurring inbreeding depression if genetic purging has not removed deleterious alleles (Byers and Waller 1999; Keller and Waller 2002).

*Argyreia versicolor* (Convolvulaceae) is an extremely rare species of morning glory endemic to Thailand (Staples and Traiperm 2010, 2017; Staples *et al.* 2021). It was first discovered in 1924 by A.F.G. Kerr, who then described and named it *Lettsomia versicolor* in 1941 (Kerr 1941). The species was later moved to the genus *Argyreia* by Khunwasi *et al.* (2005) and its name was changed to *A. versicolor*. However, after its initial discovery, this species was not seen for 94 years, until it was rediscovered in 2018 by the third author (C. Rattanamanee; Staples *et al.* 2021). Two individuals of *A. versicolor* were found in a dipterocarp forest on the campus of Burapha University in the same district that Kerr first collected it (Staples *et al.* 2021). At the same time that *A. versicolor* was rediscovered, we also discovered another rare *Argyreia* species in the same dipterocarp forest: *A. mekongensis*. *Argyreia mekongensis* was first described in 1915 (Gagnepain and Courchet 1915) and can be found not only in Thailand but also in Cambodia, Laos and Vietnam (Staples and Traiperm 2010, 2017). While *A. mekongensis* is distributed in several countries, the species is recognized as globally rare (Chaemchamron 2017). Moreover, only a couple of populations are currently known in Thailand, each with less than ten individuals, and no herbarium specimens have been collected in the past decade (P. Traiperm, pers. obs.). The causes of extreme rarity for these two species are still unknown but may be due in part to poor reproductive success (e.g. poor fertilization or germination rates) and habitat loss, as Thailand is in a biodiversity hotspot that has already lost over 90 % of its primary vegetation (Myers *et al.* 2000).

The need for information on pollination and reproduction is greatest for threatened plant species, but such information also tends to be the least accessible in the literature given the rarity of such species. For example, *A. versicolor* disappeared from the academic world for nearly 100 years, precluding studies of its pollination ecology until now. Similarly, there is barely any published information about *A. mekongensis* and nothing is known about its reproduction. Indeed, we know very little about reproduction in the genus *Argyreia* in general. There is one published study of *A. siamensis*, which found that the species is self-incompatible and relies on bee and butterfly pollinators (Jirabanjongjit *et al.* 2021). The purpose of this study is, therefore, to examine the pollination ecology of two incredibly rare species: *A. versicolor* and *A. mekongensis.* These two species were found in the same area and share several traits, such as being woody twiners and having campanulate flowers of similar size with overlapping flowering periods. Such morphological and phenological similarities suggest that they may also have similar pollinators. Specifically, the aims of this study were (i) to assess the breeding systems of the species using controlled pollination experiments and (ii) to observe floral visitors and identify potential pollinators. Such information is critical for guiding conservation efforts for these species and addresses some of the gaps in our knowledge about the understudied paleotropical genus *Argyreia*.

## Materials and Methods

### Study area and study species

We conducted this study on the campus of Bhurapa University in Sa Kaeo province, Thailand, where our two study species (*A. versicolor* and *A. mekongensis*) were found naturally occurring (13.7438228°N, 102.2885369°E). We found both species in the same area of campus, with some individuals of the two species within 10 m of each other. The study area is classified as a lowland watershed with undulating plains (Sa Kaeo Provincial Office, www.sakaeo.go.th, January 2022). Natural areas on campus are primarily covered with deciduous dipterocarp forest (A. Jirabanjongjit, pers. obs.). The local climate is tropical and seasons are governed by two monsoons, resulting in three seasons: summer, rainy and winter (Thai Meteorological Department, www.climate.tmd.go.th, January 2022). During the summer season, which spans from mid-February to mid-May, the weather typically features high temperatures (25–35 °C) accompanied by high humidity but minimal precipitation. Transitioning to the rainy season, which begins in mid-May and extends until mid-October, temperatures gradually decrease but precipitation significantly increases, especially in August and September, which is the beginning of the flowering season for our study species. During the winter, which persists from mid-October to mid-February, the weather is cooler (21–31 °C) but remains humid (Thai Meteorological Department, www.climate.tmd.go.th, January 2022).

Both study populations were extremely small. We found only two individuals of *A. versicolor* (the only two living individuals known as of this study), which were located approximately 50 m apart. We found nine individuals of *A. mekongensis*, which ranged between 500 and 1000 m apart. Both species of *Argyreia* are woody twiners found growing on wild (uncultivated) plants. At our study site, we found *A. versicolor* climbing up to the top of the tree canopy (e.g. on *Pterocarpus macrocarpus*; around 5 m from the ground) and *A. mekongensis* twinning around tree saplings and shrubs (e.g. *Dipterocarpus intricatus*, *Shorea obtusa*, and *Vietnamosasa pusilla*; around 1.5 m from the ground).

*Argyreia versicolor* is covered with hairs along all plant parts, ranging from bristly to soft (Staples and Traiperm 2010). Stems are terete and striate, and leaves are ovate with a scabrous-strigose upper side and a densely hirsute underside (Staples and Traiperm 2010). Inflorescences are axillary, containing 7–12 flowers per inflorescence (Staples and Traiperm 2010). The corolla is white, tubular-campanulate, ca. 5 cm long, and has purple-dotted limbs (Staples and Traiperm 2010). The flowers are hermaphroditic with five stamens and two stigma lobes (Staples and Traiperm 2010). Flowers are herkogamous (styles substantially longer than stamens) and pendant. Fruits are globose berries (A. Jirabanjongjit, pers. obs.). The flowering period is from August to December and fruits are mature approximately 10–12 weeks later (A. Jirabanjongjit, pers. obs.).

*Argyreia mekongensis* has hairy stems that are generally 2–4 m long (Staples and Traiperm 2010). Leaves are elliptic to broadly oblong with a sparsely strigose upper side and a tawny pubescent underside (Staples and Traiperm 2010). Inflorescences are axillary with 5–7 flowers per inflorescence (Staples and Traiperm 2010). The flowers have bracts and persistent bracteoles, which remain even after fruits are mature (Staples and Traiperm 2010). The corolla, approximately 5 cm long, is greenish white. The flowers are hermaphroditic with five stamens and two stigma lobes (Staples and Traiperm 2010). Flowers are pendant and exhibit slight herkogamy. Fruits are globose berries (Staples and Traiperm 2010). Flowers have been observed from late August through early December, and fruits are typically mature approximately 10–12 weeks later (A. Jirabanjongjit, pers. obs.).

### Mating system

To evaluate the breeding system and reproductive success of *A. versicolor* and *A. mekongensis*, we conducted controlled pollination experiments using five treatments. The open pollination treatment (flowers not manipulated in any way) tested reproductive success under natural conditions. The open-emasculated pollination treatment (anthers removed before anthesis) tested how much cross pollination occurs under natural conditions. The spontaneous autogamy treatment (flowers enclosed in fine mesh bags; hereafter referred to as the ‘closed treatment’) tested whether flowers are able to reproduce without pollinators. The hand-cross pollination treatment (flowers received xenogamous pollen transferred by researchers and were then enclosed in fine mesh bags) tested for pollen limitation. Lastly, the hand-self pollination treatment (flowers received autogamous pollen transferred by researchers and were then enclosed in fine mesh bags) tested whether flowers are self-compatible. Three months after the controlled pollination experiments, we collected mature fruits and dried them in an oven at 60 °C for 3 days. We then weighed dry fruit mass and counted the number of seeds per fruit. Seeds that were less than half the average seed size were considered inviable and not counted. In total, we used 37 flowers of *A. versicolor* from two plants (4–5 flowers per treatment per plant) in 2019 [**see Supporting Information—Table S1**]. We did not conduct pollination experiments for *A. versicolor* in 2020 given that one plant had been cut down, leaving only a single known plant, but we did look for naturally occurring fruits on our study plant, as well as for seedlings under the study plant and in the surrounding area. For *A. mekongensis*, we used 41 flowers from five plants (1–4 flowers per treatment per plant) in 2019 and 43 flowers from five plants (1–4 flowers per treatment per plant) in 2020 [**see ****Supporting Information—Table S1**].

### Pollinator observations

To observe the potential pollinators of our plant study species, we placed action cameras (Xiaomi YI Z15, Xiaomi, Beijing, China) in front of mature flower buds and set them on time-lapse mode to take a photo every 2 s throughout the entire flowering period. In 2019, we recorded 22 flowers of *A. versicolor* (from two plants across 4 days) and 25 flowers of *A. mekongensis* (from five plants across 4 days), while in 2020, we recorded 44 flowers of *A. versicolor* (from the single study plant remaining, across 8 days) and 29 flowers of *A. mekongensis* (from five plants across 7 days). We reviewed all photos, identified animals to the lowest taxonomic level possible with assistance from a local entomologist (see Acknowledgements) and calculated visitation frequency for each animal taxon. We did not collect floral visitors to avoid disturbing subsequent animal visits and to avoid damaging flowers with sweep nets. We categorized animals as ‘florivores’ if they consumed flowers or parts of flowers, ‘visitors’ if they landed on flowers but did not contact floral reproductive structures (anthers and stigmas), and ‘pollinators’ if they contacted both stigmas and anthers. Only pollinator records were used for data analysis.

### Statistical analysis

We conducted all analyses in R (version 4.1.2) (R Core Team 2022). We performed linear mixed modelling (LMM; package ‘lme4’) to investigate the effect of pollination treatment on each response variable (fruit weight and seed number). We included pollination treatment as a fixed factor and plant individual as a random factor. For *A. mekongensis*, the results of the pollination experiment were not significantly different across study years, we, therefore, pooled all data. We also conducted LMM to compare the visitation rates of pollinator taxa, where visitation rate was the response variable, animal taxon was a fixed factor, and plant individual was a random factor. We assessed the significance of each predictor using nested likelihood ratio tests (package ‘stats’). For significant factors, we used Tukey’s post hoc to compare factor levels (package ‘emmeans’). We did not perform LMM with data collected from *A. versicolor* given that our sample sizes were too small (*n* = 2 plants in 2019 and one plant in 2020).

## Results

### Mating system

**Figure 1.**
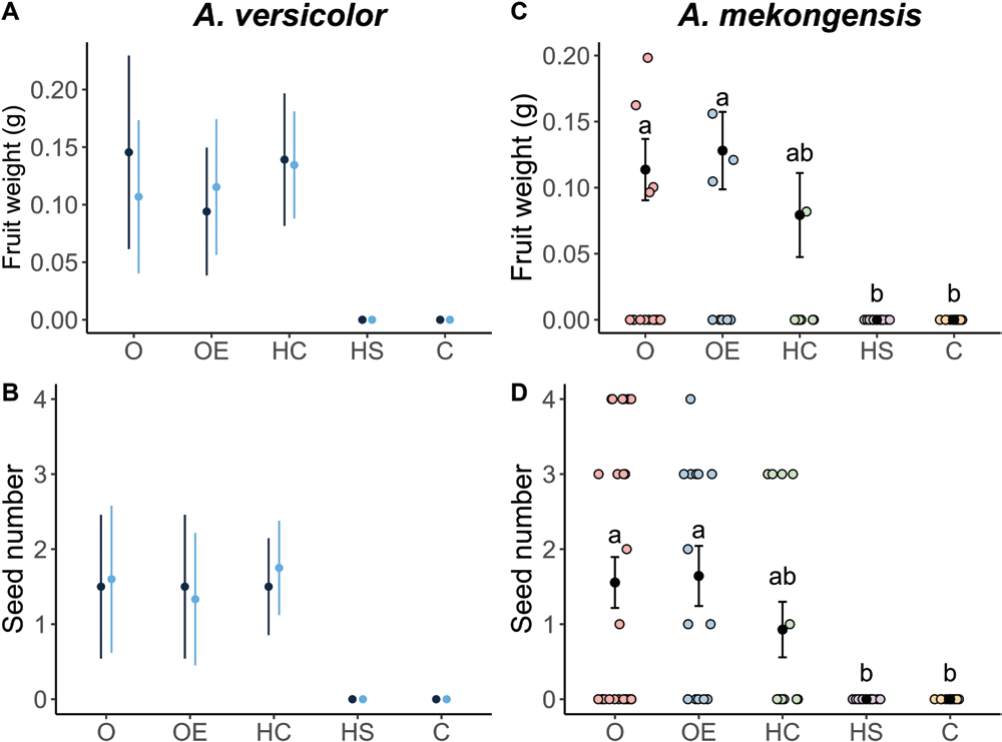
Results of controlled pollination experiments conducted on (A and B) *Argyreia versicolor* and (C and D) *Argyreia mekongensis* showing (A and C) fruit weight and (B and D) seed number for each experimental treatment (O, open pollination; OE, open emasculated; HC, hand-cross pollination; HS, hand-self pollination; and C, closed treatment). For *A. mekongensis*, black dots and error bars denote means and standard errors, while coloured jittered points show the distribution of raw data (each coloured point represents a single fruit). Treatments with different letters are significantly different (*P* < 0.05). Statistics were not performed on *A. versicolor* as there were only two known plants; dark blue = plant individual 1, light blue = plant individual 2.

The controlled pollination experiment conducted on *A. versicolor* resulted in fruit and seed set in three treatments (open, open emasculated and hand-cross pollination treatments) whereas the hand-self pollinated and closed treatments did not produce any fruit or seeds (Fig. 1A and B) [**see Supporting Information—Table S2**]. However, even though *A. versicolor* produced fruit and seed in the open, open emasculated and hand-cross pollination treatments in 2019, no seedlings were observed in the study area. No naturally occurring fruits were observed in 2020 when there was only a single remaining individual.

Similarly, the controlled pollination experiments conducted on *A. mekongensis* resulted in fruit and seed set in the open, open emasculated and hand-cross pollination treatments, whereas no fruits or seeds were found in the closed and hand-self pollination treatments (Fig. 1C and D) [**see Supporting Information—Table S2**]. LMM results revealed significant differences between treatments for both fruit weight ($X_{4}^{2}$ = 19.02, *P* < 0.001) and seed number ($X_{4}^{2}$ = 17.83, *P* < 0.005). Post hoc tests revealed that the open and open-emasculated treatments had significantly heavier fruits and set significantly more seeds than the hand-self pollinated and closed treatments (*P* < 0.05; Fig. 1C and D).

### Pollinator observations

**Figure 2.**
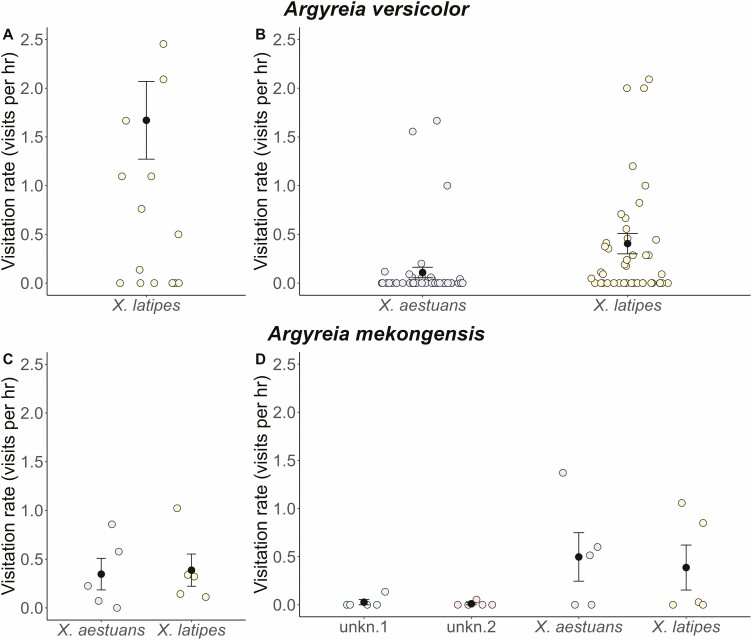
Visitation rates for the pollinators of *A. versicolor* in (A) 2019 (*n* = 2 plants) and (B) 2020 (*n* = 1 plant) and for the pollinators of *A. mekongensis* in (C) 2019 (*n* = 5 plants) and (D) 2020 (*n* = 5 plants). Black dots and error bars denote means and standard errors, while coloured jittered points show the distribution of raw data. For *A. versicolor* each coloured point represents the visitation rate to a single flower, while for *A. mekongensis* each coloured point represents the mean visitation rate across all flowers observed on the same study plant. Note: unkn.1 = small unidentified bees, unkn.2 = unidentified wasps.

**Figure 3.**
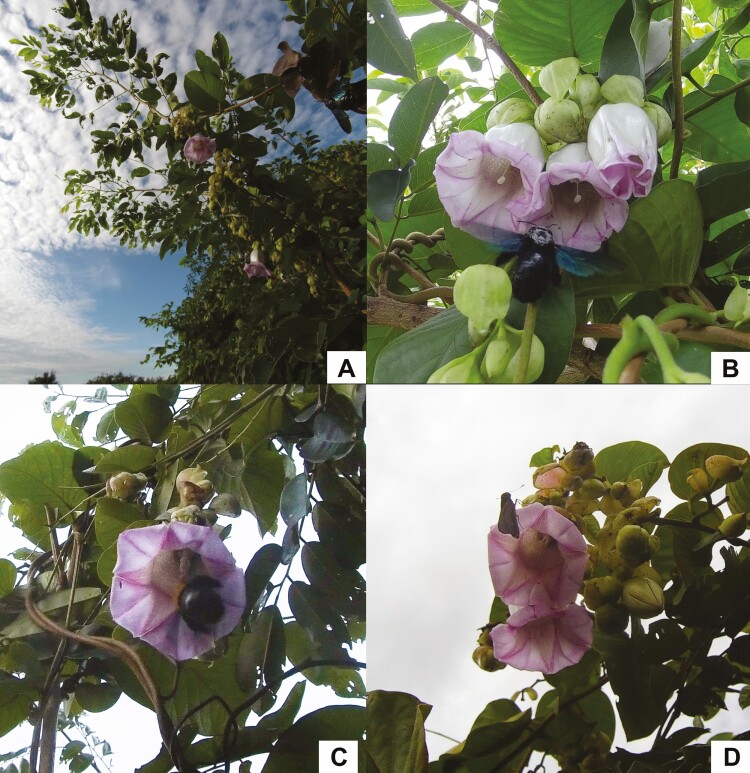
(A) *Argyreia versicolor* being visited by (B) *X. latipes* (pollinator), (C) *X. aestuans* (pollinator) and (D) Hesperiidae (floral visitor).

The time-lapse camera data revealed that *A. versicolor* was only visited by diurnal visitors (Figs 2A and B and 3) [**see Supporting Information—Table S3**]. The flowers start to open around 5:30 h, about 1 h before sunrise, and are fully open around 7:00 h. Animals were observed to start visiting flowers as early as 7:30 h and visits generally ended sometime between 16:00 h and sunset (around 18:00 h). Flowers generally wilt around 20.00 or 21.00 h. Across the 2 years of observation (approximately 177 h from 22 flowers from two plants in year 2019 and 118 h from 44 flowers from one plant in year 2020), only four animal taxa were observed visiting *A. versicolor*: females of two carpenter bee species (*Xylocopa latipes* and *Xylocopa aestuans*; (Fig. 3B and C)), a small unidentified bee species, and skipper butterflies (Hesperiidae; Fig. 3D). *Xylocopa latipes* was observed crawling into the corolla tube to forage on nectar. The large size of *X*. *latipes* ensured contact with both stamens and stigmas, and pollen was easily visible on the thorax of these native carpenter bees (Fig. 3B). The foraging behaviour of *X*. *aestuans* (Fig. 3C) was very similar to that of *X*. *latipes*, but it was only observed in 2020 and visited flowers less frequently than did *X. latipes* (Fig. 2A and B). Two groups of floral visitors were occasionally observed: small unidentified bees landed on the stamens but never contacted the stigmas, and skipper butterflies landed on the petals but never entered the corolla tube (Fig. 2D). In 2019, we only observed *X. latipes* as likely pollinators (Fig. 2A). In 2020, we observed two likely pollinator species, *X. latipes* and *X. aestuans* (Fig. 2B).

**Figure 4.**
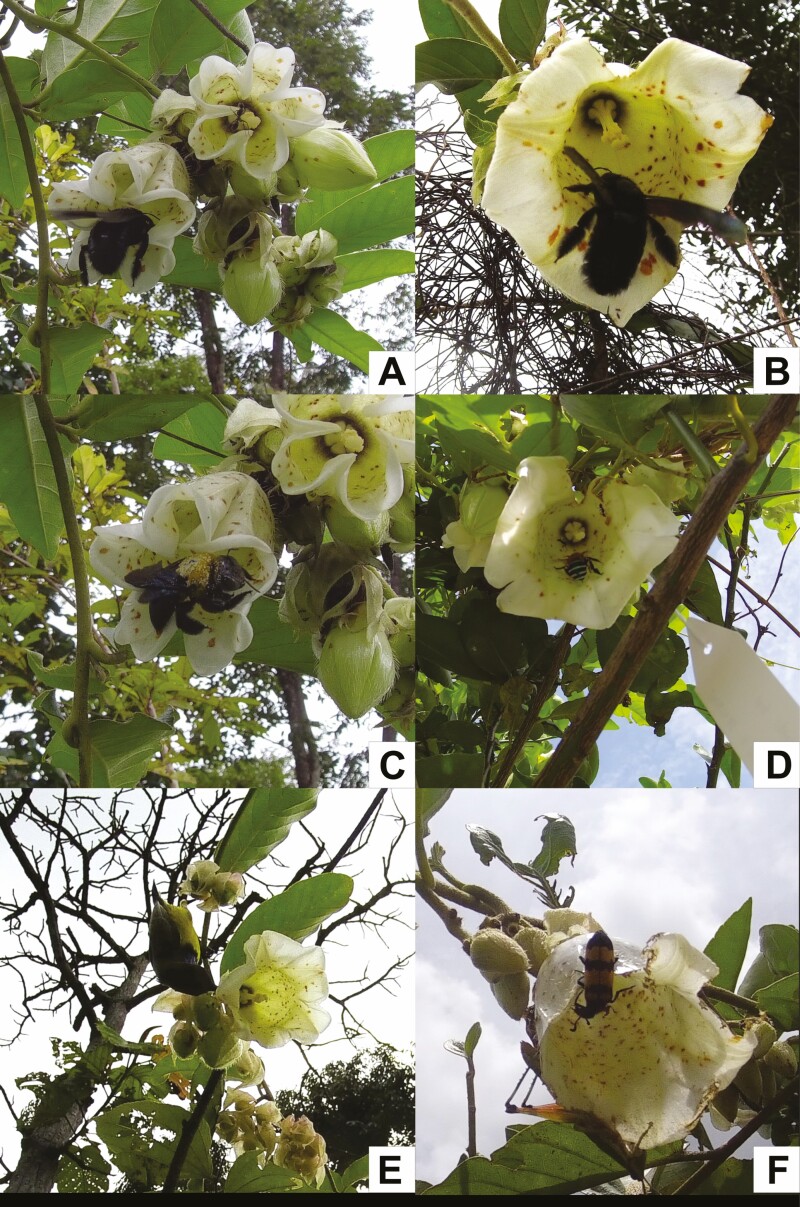
*Argyreia mekongensis* being visited by its two main pollinators (A and B) *X. latipes* and (C) *X. aestuans*, two floral visitors (D) *Amegilla* sp. and (E) *C. jugularis*, and two florivores (F) *M. phalerata* (upper edge of corolla) and a grasshopper (lower edge of corolla).

The flowers of *A. mekongensis* were also only visited by diurnal visitors (Figs 2C and D and 4) [**see Supporting Information—Table S3**], despite anthesis spanning over 36 h. Flowers start to open around 5:00 h, are fully open around 7:00-8:00 h, and remain open until the evening of the following day. Across the 2 years of observation (approximately 74 h from 25 flowers from five plants in 2019 and 135 h from 29 flowers from five plants in 2020), we observed diverse animal taxa visiting the flowers. Females of two carpenter bee species (*X. aestuans* and *X. latipes*; Fig. 4A–C) were common visitors, visiting flowers throughout the day until sunset. Both species crawled inside the corolla to forage on nectar and always contacted the anthers and stigmas, with pollen visibly abundant on their fuzzy thorax (Fig. 4C). Blue-banded bees (*Amegilla* sp.; Fig. 4D) visited flowers in the morning until noon. *Amegilla* bees were observed to land inside the corolla and crawl to the nectary at the corolla base. However, given their small size relative to the corolla tube, blue-banded bees did not contact either anthers or stigmas and were, therefore, classified as floral visitors. Small unidentified bees were also observed visiting flowers, and while they are even smaller than *Amegilla* bees, they generally foraged on pollen, not nectar, and were, therefore, classified as potential pollinators given that they contacted both anthers and stigmas during foraging. Multiple grasshopper taxa (Acrididae; Fig. 4F) visited flowers, consuming the petals and occasionally the stigmas and anthers. Skipper butterflies (Hesperiidae) were categorized as visitors given that they only landed on floral petals and never entered the corolla tube. Blister beetles (*Mylabris phalerata*; Fig. 4F) were occasionally observed visiting the flowers of *A. mekongensis*. They typically visited in groups of 2–10 individuals and were observed foraging on the petals, anthers and stigmas. While blister beetles were observed with pollen on their bodies, their foraging activity caused bruising to the anthers and stigmas, we, therefore, categorized them as florivores due to their destructive foraging behaviour. Their presence on flowers also appeared to deter visits by *X. latipes* and *X. aestuans*, which did not land on flowers occupied by *M. phalerata*. Visitation by sunbirds (Nectariniidae) was uncommon and cameras captured bird visits only in 2020; *Cinnyris jugularis*, the olive-backed sunbird (Fig. 4E), was observed to perch near the corolla base and use their beak to pierce the corolla base to rob nectar. Unidentified wasps were also observed a few times and would usually walk on stamens, contacting both stigmas and anthers in the process, so they were, therefore, categorized as potential pollinators.

In 2019, we observed two potential pollinator species visiting *A. mekongensis* flowers, *X. latipes* and *X. aestuans* (Fig. 2C), and their visitation rates were not significantly different ($X_{1}^{2}$ = 1.10, *P* = 0.295). In 2020, we observed *X. latipes*, *X. aestuans*, small unidentified bees and unidentified wasps as potential pollinators (Fig. 2D), and differences in their visitation rates were marginally significant ($X_{3}^{2}$ = 7.62, *P* = 0.055).

## Discussion

### Mating systems of *Argyreia versicolor* and *Argyreia mekongensis*

Our controlled pollination experiments revealed that both *A. versicolor* and *A. mekongensis* are completely self-incompatible and that pollinators are essential for their reproduction. The findings of this study are similar to those of a sister species, *Argyreia siamensis*, which is also completely self-incompatible (Jirabanjongjit *et al.* 2021). Self-incompatibility has also been reported in other species in the Convolvulaceae, such as *Ipomoea pandurata* (Stucky and Beckmann 1982), *I. pes-caprae* (Devall and Thein 1992), *I. cairica* (Jia *et al.* 2007) and *I. bahiensis* (Pick and Schlindwein 2011). However, the Convolvulaceae family is highly diverse, and other mating systems are common, including self-compatible (Pick and Schlindwein 2011; Delgado-Dávila *et al.* 2016) and mixed mating systems (Chang and Rausher 1999; McMullen 2009; Wright *et al.* 2012). Self-incompatibility is well-known as an important and prevalent mechanism that promotes outcrossing in angiosperms (Whitehouse 1950; Richards 1997; Allen and Hiscock 2008; Narayanapur *et al.* 2018) and the results of our controlled pollination experiments, coupled with the findings from our pollinator observations, indicate that both *A. versicolor* and *A. mekongensis* are reliant on pollinators to promote outcrossing. Interestingly, while both species are herkogamous to different degrees, their flowers are pendant and autogamous pollen likely falls on the stigmas, which suggests a genetic basis for their self-incompatibility rather than a physical mechanism. However, intraspecific variation in self-compatibility has been reported in the Convolvulaceae (Hassa *et al.* 2023), so examination of other populations, if possible, would improve our understanding of their mating systems.

While *A. mekongensis* was found to be self-incompatible, it does not appear to experience quantitative pollen limitation, as there were no significant differences between the open, open emasculated and hand-cross pollinated treatments in terms of both fruit and seed set. In general, self-incompatible species tend to be more likely to experience pollen limitation than self-compatible species (Burd 1994; Larson and Barrett 2000; Knight *et al.* 2005). Endemic species found in highly diverse areas are especially impacted (Myers *et al.* 2000; Vamosi *et al.* 2006; Alonso *et al.* 2010) due to a lack of compatible pollen and/or inadequate pollinator visitation (de Arroyo 1976; Bullock *et al.* 1989). For example, pollen limitation due to insufficient pollinator visitation has been reported in *Ipomoea habeliana* (McMullen 2009), while pollen limitation due to inadequate pollen quality has be reported in *Ipomoea pes-caprae* (Raju *et al.* 2014). The lack of observed quantitative pollen limitation in this study is somewhat surprising given that species with small population sizes, like *A. mekongensis*, often experience pollen limitation due to low pollinator visitation rates, low pollen deposition (Regal 1982; Whitehead 1983; Feinsinger *et al.* 1991; Fausto *et al.* 2001) and/or frequent intra-plant pollinator movement (Klinkhamer and de Jong 1990; Franceschinelli and Bawa 2000; Mustajärvi *et al.* 2001; Iwaizumi and Sakai 2004). One factor that may contribute to the reproductive success of *A. mekongensis*, in spite of its small population size, is low competition for pollinators, as there are few other plant species in bloom in our study area during the flowering period of *A. mekongensis*. Another important factor may be high pollinator fidelity, as *A. mekongensis* appears to be pollinated almost exclusively by carpenter bees, which have been shown to demonstrate high floral fidelity (Araújo *et al.* 2021).

The pollination experiment results of *A. versicolor* were similar to those of *A. mekongensis,* producing fruits and seeds in three of the five treatments. However, no seedlings were ever observed during regular visits to the study area throughout the study and in the months following the fruiting season (C. Rattanamanee, pers. obs.). It is not clear why germination was not successful. The results of our pollinator observations, and the fact that naturally pollinated flowers set as many fruits and seeds as hand-cross pollinated flowers, indicate that inadequate pollinator visitation is not the cause. One possible explanation is seed or fruit predation, as was proposed for *A. siamensis* (Jirabanjongjit *et al.* 2021), although we did not observe any instances of such predation in our study area. A second possible explanation is that the two individuals of *A. versicolor* examined in this study may have been siblings or close relatives. The two individuals of this self-incompatible species may have been genetically different enough for ovule formation to begin but may have been too similar for normal development to be completed. Supporting this conjecture, we attempted to germinate *A. versicolor* seeds in a greenhouse but were not successful. Such circumstances have been previously reported. For example, Collevatti *et al.* (2009) found aborted seeds from self and cross pollination in *Caryocar brasiliense*, and they concluded that seed abortion resulting from cross pollination was caused by biparental inbreeding depression due to the pollen donor being closely related to the mother tree. Tragically, only two living individuals of *A. versicolor* were known to exist when we began this study in 2019, and one individual was felled before the start of the 2020 flowering season. The remaining *A. versicolor* individual in our study area did not produce any fruits in 2020, despite being visited by *Xylocopa* carpenter bees (same as the previous year), and no other living individuals are currently known for this endemic species on the verge of extinction. Multiple searches at our study site and the surrounding areas have not revealed any other individuals. Moreover, there have been no references in the literature or herbarium records of this species since the type specimen, thus, finding other individuals will rely on chance encounters by knowledgeable botanists.

### Pollinators of *Argyreia versicolor* and *Argyreia mekongensis*

Our pollinator observations indicate that both *A. versicolor* and *A. mekongensis* are pollinated by carpenter bees. Previous studies have generally reported that Convolvulaceae species are mainly pollinated by bees (Galetto and Bernadello 2004; Maimoni-Rodella and Yanagizawa 2007; Pick and Schlindwein 2011; Hassa *et al.* 2023) or by bees and butterflies (Hassa *et al.* 2020; Jirabanjongjit *et al.* 2021) or by hawkmoths (Willmott and Burquez 1996; McMullen 2009; Chitchak *et al.* 2022). Pollination studies of *Argyreia* are scarce compared to sister genera such *Ipomoea*, but Chitchak *et al.* (2018) did report that *Xylocopa nasalis* visits *Argyreia gyrobracteata* and receives pollen on its head. Other reports of carpenter bees visiting morning glory flowers are rare. A study around the Gulf of Mexico found that *Ipomoea pes-caprae* flowers were primarily pollinated by two species of *Xylocopa* (*X. brasilianorum* and *X. strandi*; Devall and Thien 1989), while *I. pes-caprae* flowers in India were visited by several bee species, including *X. latipes* and *Xylocopa pubescens* (Raju *et al.* 2014). Studies of *I. cairica* have reported diverse pollinators, but one study in China found a species of *Xylocopa* to be an effective pollinator (Jia *et al.* 2007). In contrast, Keeler (1977) reported *Xylocopa frontalis* as a nectar robber of *I. carnea* in Costa Rica. This species has a narrow corolla tube that carpenter bees are unable to enter, but the bees were observed piercing the base of the corolla to gain access to nectar (Keeler 1977).

While reports of carpenter bee pollination in the Convolvulaceae family are uncommon, *A. versicolor* and *A. mekongensis* have several traits that correspond with their large bee pollinators. The flowers of both species are bell-shaped and similar in size. The wide corolla tubes of both *Argyreia* species are able to accommodate their large carpenter bee pollinators, as both *Xylocopa* species were observed landing just inside the entrance of the corolla tube and then crawling inside to forage from the nectary located at the base of the corolla. The anthers and stigmas of both *Argyreia* species are also perfectly positioned to contact the thorax of their *Xylocopa* pollinators, and precise pollen placement (sensu Stewart *et al.* 2022) is common among carpenter bee-pollinated flowers (Momose *et al.* 1998). The flowers of both species are also robust and able to withstand rough handling by their large pollinators (Raju and Rao 2006). Moreover, *Xylocopa* species have been reported to favour purplish-white flowers and creamy white flowers (Raju and Rao 2006), as seen in our study species.

The fact that *A. versicolor* and *A. mekongensis* were both visited almost exclusively by *Xylocopa* bees over both study years indicates that these morning glory species are specialists in terms of their pollinators. On the other end of the interaction, *Xylocopa* have been shown to be generalist foragers at the species level, visiting a large number of plant species (Stewart *et al.* 2018; Araújo *et al.* 2021). Memmott *et al.* (2004) examined plant pollinator networks and found that specialist species tend to interact with generalist species, as observed in our study system, which helps buffer against species extinctions. Having common and generalist pollinators is undoubtedly beneficial for the extremely rare *A. versicolor* and *A. mekongensis*, especially since *Xylocopa* bees, while generalist foragers at the species level, have been shown to be specialists with high floral fidelity at the individual level (Araújo *et al.* 2021), which can reduce disadvantageous interspecific pollen transfer between co-occurring species (Brosi 2016).

### Conclusions

Our findings reveal that *A. versicolor* and *A. mekongensis* are both self-incompatible and completely dependent on pollinators for reproduction. We also found that both species rely on carpenter bees (*X. latipes* and *X. aestuans*) for pollination. Reports of carpenter bee pollination in the Convolvulaceae are uncommon, but our two study species exhibit several floral traits that correspond with their large bee pollinators, such as a wide corolla tube as well as anthers and stigmas that are perfectly positioned to contact the bee’s thorax. Such information about mating systems and pollinators is important for plant conservation, especially for species that are endemic, rare and comprise small populations.

Tragically, conservation efforts may be too late to protect *A. versicolor*. When the species was rediscovered in 2018, nearly 100 years after its last sighting, we knew of two individuals. In 2020, one of these individuals was felled, leaving a single remaining known individual. This remaining individual has not set fruit since, which indicates that there are no conspecific individuals in the near vicinity. We attempted to vegetatively propagate *A. versicolor* from cuttings, which successfully established roots in the greenhouse but did not survive following transplant to the wild. Moreover, given that *A. versicolor* is self-incompatible, vegetative propagation is not a long-term solution to helping the species recover. The conservation status of *A. mekongensis* has not yet been assessed, but given that only two populations are currently known in Thailand, and that populations generally harbour only 3–9 individuals, immediate steps should be taken to protect *A. mekongensis* and other similarly rare plant species. In our study area, individuals of *A. mekongensis* produced fruits with viable seeds, but population recruitment remained negligible. One potential explanation is a late-acting biparental inbreeding depression, evident in later life stages such as seedling survival. Considering the significantly reduced population size, this mechanism driven by inflated kinship between individuals may be plausible. For self-incompatible species with small population sizes, such as *A. mekongensis*, human-assisted gene flow (e.g. transplanting seedlings across populations) can help increase intra-population genetic diversity and improve plant reproductive success. Obtaining genetic information would improve our understanding of intra- and inter-population diversity and dispersal patterns, allowing a genetic management strategy based on kinship to be formulated. Moreover, habitat loss continues to be a major threat to rare plant species worldwide. The natural habitat of *A. versicolor* and *A. mekongensis* is often destroyed due to deforestation and swidden agriculture practiced by locals, thus, habitat protection is of utmost importance for both plants and the pollinators they depend on.

## Supporting Information

The following additional information is available in the online version of this article –

Table S1. Summary of pollination experiment sample sizes.

Table S2. Raw data from pollination experiments.

Table S3. Raw data from pollinator observations.

plae001_suppl_Supplementary_Tables_S1-S3

## Data Availability

All data can be found in the supporting information files.

## References

[CIT0002] Allen AM , Hiscock SJ. 2008. Evolution and phylogeny of self-incompatibility systems in angiosperms. In: Franklin-Tong, VE, ed. Self-incompatibility in flowering plants. Berlin: Springer Berlin, Heidelberg, 73–101. doi: 10.1007/978-3-540-68486-2

[CIT0003] Alonso C , Vamosi JC, Knight TM, Steets JA, Ashman TL. 2010. Is reproduction of endemic plant species particularly pollen limited in biodiversity hotspots? Oikos 119:1192–1200.

[CIT0004] Araújo TN , Pires LP, Meireles DAL, Augusto SC. 2021. Individual‐resource network between *Xylocopa* bees and plant resources: generalist species, specialist individuals? Ecological Entomology 46:1273–1282.

[CIT0006] Bond WJ. 1994. Do mutualisms matter? Assessing the impact of pollinator and disperser disruption on plant extinction. Philosophical Transactions of the Royal Society of London, Series B: Biological Sciences 344:83–90.

[CIT0007] Brosi BJ. 2016. Pollinator specialization: from the individual to the community. New Phytologist 210:1190–1194. 27038018 10.1111/nph.13951

[CIT0008] Bullock SH , Del Rio CM, Ayala R. 1989. Bee visitation rates to trees of *Prockia crucis* differing in flower number. Oecologia 78:389–393. 28312586 10.1007/BF00379114

[CIT0009] Burd M. 1994. Bateman’s principle and plant reproduction: the role of pollen limitation in fruit and seed set. The Botanical Review 60:83–139.

[CIT0010] Byers DL , Waller DM. 1999. Do plant populations purge their genetic load? Effects of population size and mating history on inbreeding depression. Annual Review of Ecology and Systematics 30:479–513.

[CIT0011] Campbell DR , Motten AF. 1985. The mechanism of competition for pollination between two forest herbs. Ecology 66:554–563.

[CIT0012] Chaemchamrun W. 2017. Threatened plants in Thailand. Bangkok: Forest Herbarium.

[CIT0013] Chang SM , Rausher MD. 1999. The role of inbreeding depression in maintaining the mixed mating system of the common morning glory, *Ipomoea purpurea*. Evolution 53:1366–1376. 28565568 10.1111/j.1558-5646.1999.tb05401.x

[CIT0014] Chitchak N , Traiperm P, Staples G, Rattanakrajang P, Sumanon P. 2018. Species delimitation of some *Argyreia* (Convolvulaceae) using phenetic analyses: insights from leaf anatomical data reveal a new species. Botany 96:217–233.

[CIT0015] Chitchak N , Stewart AB, Traiperm P. 2022. Functional ecology of external secretory structures in *Rivea ornata* (Roxb.) Choisy (Convolvulaceae). Plants (Basel, Switzerland) 11:2068. 35956546 10.3390/plants11152068 PMC9370475

[CIT0016] Collevatti RG , Estolano R, Garcia SF, Hay JD. 2009. Seed abortion in the bat pollinated Neotropical tree species, *Caryocar brasiliense* (Caryocaraceae). Botany 87:1110–1115.

[CIT0017] de Arroyo MTK. 1976. Geitonogamy in animal pollinated tropical angiosperms: a stimulus for the evolution of self‐incompatibility. Taxon 25:543–548.

[CIT0018] Delgado‐Dávila R , Martén‐Rodríguez S, Huerta‐Ramos G. 2016. Variation in floral morphology and plant reproductive success in four *Ipomoea* species (Convolvulaceae) with contrasting breeding systems. Plant Biology 18:903–912. 27634630 10.1111/plb.12507

[CIT0019] Delnevo N , van Etten EJ, Byrne M, Stock WD. 2019. Floral display and habitat fragmentation: effects on the reproductive success of the threatened mass‐flowering *Conospermum undulatum* (Proteaceae). Ecology and Evolution 9:11494–11503. 31641488 10.1002/ece3.5653 PMC6802041

[CIT0020] Devall MS , Thien LB. 1989. Factors influencing the reproductive success of *Ipomoea pes‐caprae* (Convolvulaceae) around the Gulf of Mexico. American Journal of Botany 76:1821–1831.

[CIT0021] Devall MS , Thien LB. 1992. Self-incompatibility in *Ipomoea pes-caprae* (Convolvulaceae). American Midland Naturalist 128:22–29.

[CIT0023] Fausto Jr JA , Eckhart VM, Geber MA. 2001. Reproductive assurance and the evolutionary ecology of self‐pollination in *Clarkia xantiana* (Onagraceae). American Journal of Botany 88:1794–1800. 21669612

[CIT0024] Feinsinger P , Tiebout III HM, Young BE. 1991. Do tropical bird‐pollinated plants exhibit density‐dependent interactions? Field experiments. Ecology 72:1953–1963.

[CIT0025] Franceschinelli EV , Bawa KS. 2000. The effect of ecological factors on the mating system of a South American shrub species (*Helicteres brevispira*). Heredity 84 ( Pt 1):116–123. 10692018 10.1046/j.1365-2540.2000.00636.x

[CIT0026] Gagnepain F , Courchet L. 1915. Convolvulacées Asiatiques Nouvelles. Notulae Systematicae 3:134–135.

[CIT0027] Galetto L , Bernardello G. 2004. Floral nectaries, nectar production dynamics and chemical composition in six *Ipomoea* species (Convolvulaceae) in relation to pollinators. Annals of Botany 94:269–280. 15229123 10.1093/aob/mch137 PMC4242162

[CIT0028] Gargano D , Gullo T, Bernardo L. 2009. Do inefficient selfing and inbreeding depression challenge the persistence of the rare *Dianthus guliae* Janka (Caryophyllaceae)? Influence of reproductive traits on a plant’s proneness to extinction. Plant Species Biology 24:69–76.

[CIT0029] Hassa P , Traiperm P, Stewart AB. 2020. Pollinator visitation and female reproductive success in two floral color morphs of *Ipomoea aquatica* (Convolvulaceae). Plant Systematics and Evolution 306:1–11.

[CIT0030] Hassa P , Traiperm P, Stewart AB. 2023. Compatibility systems and pollinator dependency in morning glory species (Convolvulaceae). BMC Plant Biology 23:432. 37715144 10.1186/s12870-023-04437-y PMC10503090

[CIT0031] Iwaizumi MG , Sakai S. 2004. Variation in flower biomass among nearby populations of *Impatiens textori* (Balsaminaceae): effects of population plant densities. Canadian Journal of Botany 82:563–572.

[CIT0032] Jia X , Li X, Dan Y, Lu G, Wang Y. 2007. Pollination biology of an invasive weed *Ipomoea cairica*. Biodiversity Science 15:592.

[CIT0033] Jirabanjongjit A , Traiperm P, Sando T, Stewart AB. 2021. Pollination and floral biology of a rare morning glory species endemic to Thailand, *Argyreia siamensis*. Plants (Basel, Switzerland) 10:2402. 34834765 10.3390/plants10112402 PMC8623002

[CIT0034] Keeler KH. 1977. The extrafloral nectaries of *Ipomoea carnea* (Convolvulaceae). American Journal of Botany 64:1182–1188.

[CIT0035] Keller LF , Waller DM. 2002. Inbreeding effects in wild populations. Trends in Ecology & Evolution 17:230–241.

[CIT0036] Kerr AFG. 1941. Contributions to the Flora of Thailand. Additamentum LIV. Bulletin of Miscellaneous Information (Royal Botanic Gardens, Kew): 1941:8–21.

[CIT0037] Khunwasi C , Songkhla BN, Traiperm P, Staples GW. 2005. Four new combinations in *Argyreia* Lour. (Convolvulaceae). Thai Forest Bulletin (Botany) 33:42–43.

[CIT0038] Klinkhamer PG , de Jong TJ. 1990. Effects of plant size, plant density and sex differential nectar reward on pollinator visitation in the protandrous *Echium vulgare* (Boraginaceae). Oikos 57:399–405. doi:10.2307/3565970

[CIT0039] Knight TM , Steets JA, Vamosi JC, Mazer SJ, Burd M, Campbell DR, Dudash MR, Johnston MO, Mitchell RJ, Ashman TL. 2005. Pollen limitation of plant reproduction: pattern and process. Annual Review of Ecology, Evolution, and Systematics 36:467–497.

[CIT0040] Kunin WE. 1992. Density and reproductive success in wild populations of *Diplotaxis erucoides* (Brassicaceae). Oecologia 91:129–133. 28313384 10.1007/BF00317251

[CIT0041] Kunin WE. 1993. Sex and the single mustard: population density and pollinator behavior effects on seed‐set. Ecology 74:2145–2160.

[CIT0042] Kwak MM , Jennersten O. 1991. Bumblebee visitation and seedset in *Melampyrum pratense* and *Viscaria vulgaris*: heterospecific pollen and pollen limitation. Oecologia 86:99–104. 28313164 10.1007/BF00317395

[CIT0043] Larson BM , Barrett SC. 2000. A comparative analysis of pollen limitation in flowering plants. Biological Journal of the Linnean Society 69:503–520.

[CIT0044] Levin DA. 1972. Competition for pollinator service: a stimulus for the evolution of autogamy. Evolution 26:668–669. 28563360 10.1111/j.1558-5646.1972.tb01972.x

[CIT0045] Maimoni-Rodella RCS , Yanagizawa YANP. 2007. Floral biology and breeding system of three *Ipomoea* weeds. Planta Daninha 25:35–42.

[CIT0046] McMullen CK. 2009. Pollination biology of a night-flowering Galápagos endemic, *Ipomoea habeliana* (Convolvulaceae). Botanical Journal of the Linnean Society 160:11–20.

[CIT0047] Memmott J , Waser NM, Price MV. 2004. Tolerance of pollination networks to species extinctions. Proceedings Biological Sciences 271:2605–2611. doi:10.1098/rspb.2004.2909 15615687 PMC1691904

[CIT0048] Momose K , Yumoto T, Nagamitsu T, Kato M, Nagamasu H, Sakai S, Harrison RD, Itioka T, Hamid AA, Inoue T. 1998. Pollination biology in a lowland dipterocarp forest in Sarawak, Malaysia. I. Characteristics of the plant‐pollinator community in a lowland dipterocarp forest. American Journal of Botany 85:1477–1501. 21684899

[CIT0049] Mosquin T. 1971. Competition for pollinators as a stimulus for the evolution of flowering time. Oikos 22:398–402.

[CIT0050] Mustajärvi K , Siikamäki P, Rytkönen S, Lammi A. 2001. Consequences of plant population size and density for plant-pollinator interactions and plant performance. Journal of Ecology 89:80–87.

[CIT0051] Myers N , Mittermeier RA, Mittermeier CG, Da Fonseca GA, Kent J. 2000. Biodiversity hotspots for conservation priorities. Nature 403:853–858. 10706275 10.1038/35002501

[CIT0052] Narayanapur VB , Suma B, Minimol JS. 2018. Self-incompatibility: a pollination control mechanism in plants. International Journal of Plant Sciences 13:201–212.

[CIT0053] Pick RA , Schlindwein C. 2011. Pollen partitioning of three species of Convolvulaceae among oligolectic bees in the Caatinga of Brazil. Plant Systematics and Evolution 293:147–159.

[CIT0054] R Core Team 2022. R: A language and environment for statistical computing. Vienna, Austria: R Foundation for Statistical Computing. https://www.R-project.org/

[CIT0055] Raju AS , Rao SP. 2006. Nesting habits, floral resources and foraging ecology of large carpenter bees (*Xylocopa latipes* and *Xylocopa pubescens*) in India. Current Science 90:1210–1217.

[CIT0056] Raju AS , Raju PS, Ramana KV. 2014. Melittophily and malacophily in *Ipomoea pes-caprae* (Convolvulaceae). TAPROBANICA: The Journal of Asian Biodiversity 6:90–99.

[CIT0057] Rathcke B. 1983. Competition and facilitation among plants for pollination. In: Real L. ed. Pollination biology. New York: Academic Press, 305–329. doi: 10.1016/B978-0-12-583980-8.50019-3

[CIT0058] Regal PJ. 1982. Pollination by wind and animals: ecology of geographic patterns. Annual Review of Ecology and Systematics 13:497–524.

[CIT0059] Richards AJ. 1997. Plant breeding systems. London: Chapman & Hall.

[CIT0060] Schoen DJ , Lloyd DG. 1992. Self-and cross-fertilization in plants. III. Methods for studying modes and functional aspects of self-fertilization. International Journal of Plant Sciences 153:381–393.

[CIT0061] Staples GW , Traiperm P. 2010. *Argyreia* Lour. In: Santisuk T., Larsen K.. eds. Flora of Thailand, *Vol.* 10(3). Bangkok: Royal Forest Department, 337–371.

[CIT0062] Staples GW , Traiperm P. 2017. A nomenclatural review of *Argyreia* (Convolvulaceae). Taxon 66:445–477.

[CIT0063] Staples GW , Chitchak N, Kochaiphat P, Rattamanee C, Rattanakrajang P, Traiperm P. 2021. Convolvulaceae in the Flora of Thailand: Addenda, Corrigenda and Emendanda, I. Thai Forest Bulletin (Botany) 49:88–101.

[CIT0064] Stewart AB , Sritongchuay T, Teartisup P, Kaewsomboon S, Bumrungsri S. 2018. Habitat and landscape factors influence pollinators in a tropical megacity, Bangkok, Thailand. PeerJ 6:e5335. 30042902 10.7717/peerj.5335 PMC6055598

[CIT0065] Stewart AB , Diller C, Dudash MR, Fenster CB. 2022. Pollination‐precision hypothesis: support from native honey bees and nectar bats. New Phytologist 235:1629–1640. 35194792 10.1111/nph.18050

[CIT0066] Stucky JM , Beckmann RL. 1982. Pollination biology, self‐incompatibility, and sterility in *Ipomoea pandurata* (L.) G.F.W.Meyer (Convolvulaceae). American Journal of Botany 69:1022–1031.

[CIT0067] Thomson JD , Andrews BJ, Plowright RC. 1981. The effect of a foreign pollen on ovulae development in *Diervilla lonicera* (Caprifoliaceae). New Phytologist 90:777–783.

[CIT0068] Vamosi JC , Knight TM, Steets JA, Mazer SJ, Burd M, Ashman TL. 2006. Pollination decays in biodiversity hotspots. Proceedings of the National Academy of Sciences of the United States of America 103:956–961. 16418284 10.1073/pnas.0507165103 PMC1347978

[CIT0069] Waser NM. 1978. Interspecific pollen transfer and competition between co-occurring plant species. Oecologia 36:223–236. 28309130 10.1007/BF00349811

[CIT0070] Whitehead DR. 1983. Wind pollination: some ecological and evolutionary perspectives. In: Real L, ed. Pollination biology. New York: Academic Press, 97–108. doi: 10.1016/B978-0-12-583980-8.50012-0

[CIT0071] Whitehouse HL. 1950. Multiple-allelomorph incompatibility of pollen and style in the evolution of the angiosperms. Annals of Botany 14:199–216.

[CIT0072] Wilcock C , Neiland R. 2002. Pollination failure in plants: why it happens and when it matters. Trends in Plant Science 7:270–277. 12049924 10.1016/s1360-1385(02)02258-6

[CIT0073] Willmott AP , Burquez A. 1996. The pollination of *Merremia palmeri* (Convolvulaceae): can hawk moths be trusted? American Journal of Botany 83:1050–1056.

[CIT0074] Wright MA , Lanni MD, Costea M. 2012. Diversity and evolution of pollen-ovule production in *Cuscuta* (dodders, Convolvulaceae) in relation to floral morphology. Plant Systematics and Evolution 298:369–389.

