# Supplementary material for: Near extinct Argyreia versicolor and rare Argyreia mekongensis are dependent on carpenter bee pollinators
Source: AoB Plants. 2024 Jan 17;16(2):plae001. doi: 10.1093/aobpla/plae001 (PMC10862652; doi:10.1093/aobpla/plae001)
Supplement: plae001_suppl_Supplementary_Tables_S1-S3 [file plae001_suppl_supplementary_tables_s1-s3.docx]

## Near extinct *Argyreia versicolor* and rare *Argyreia mekongensis* are dependent on carpenter bee pollinators

## Supporting Information

**Table S1. Summary of pollination experiment sample sizes**

| **Plant Species** | **Year** | **Treatment** | **Total # Plants** | **Total # Flowers** |
| --- | --- | --- | --- | --- |
| A. versicolor | 2019 | O | 2 | 9 |
| A. versicolor | 2019 | OE | 2 | 7 |
| A. versicolor | 2019 | HC | 2 | 8 |
| A. versicolor | 2019 | HS | 2 | 8 |
| A. versicolor | 2019 | C | 2 | 5 |
| A. mekongensis | 2019 | O | 5 | 16 |
| A. mekongensis | 2019 | OE | 5 | 7 |
| A. mekongensis | 2019 | HC | 5 | 6 |
| A. mekongensis | 2019 | HS | 5 | 6 |
| A. mekongensis | 2019 | C | 5 | 6 |
| A. mekongensis | 2020 | O | 5 | 11 |
| A. mekongensis | 2020 | OE | 5 | 7 |
| A. mekongensis | 2020 | HC | 5 | 8 |
| A. mekongensis | 2020 | HS | 5 | 8 |
| A. mekongensis | 2020 | C | 5 | 9 |

Note: O = open pollination, OE = open emasculation treatment, HC = hand-cross pollination treatment, HS = hand-self pollination treatment, C = closed (spontaneous autogamy) treatment

**Table S2. Raw data from pollination experiments.**

| **Plant Species** | **Year** | **Plant ID** | **Treatment** | **Fruit weight (g)** | **Seed number** |
| --- | --- | --- | --- | --- | --- |
| A. versicolor | 2019 | 1 | O | 0.2921 | 4 |
| A. versicolor | 2019 | 1 | O | 0 | 0 |
| A. versicolor | 2019 | 1 | O | 0 | 0 |
| A. versicolor | 2019 | 1 | O | 0.29 | 2 |
| A. versicolor | 2019 | 2 | O | 0.305 | 4 |
| A. versicolor | 2019 | 2 | O | 0.2294 | 4 |
| A. versicolor | 2019 | 2 | O | 0 | 0 |
| A. versicolor | 2019 | 2 | O | 0 | 0 |
| A. versicolor | 2019 | 2 | O | 0 | 0 |
| A. versicolor | 2019 | 1 | OE | 0 | 0 |
| A. versicolor | 2019 | 1 | OE | 0.1598 | 2 |
| A. versicolor | 2019 | 1 | OE | 0.2164 | 4 |
| A. versicolor | 2019 | 1 | OE | 0 | 0 |
| A. versicolor | 2019 | 2 | OE | 0.1942 | 1 |
| A. versicolor | 2019 | 2 | OE | 0 | 0 |
| A. versicolor | 2019 | 2 | OE | 0.1518 | 3 |
| A. versicolor | 2019 | 1 | C | 0 | 0 |
| A. versicolor | 2019 | 1 | C | 0 | 0 |
| A. versicolor | 2019 | 1 | C | 0 | 0 |
| A. versicolor | 2019 | 1 | C | 0 | 0 |
| A. versicolor | 2019 | 2 | C | 0 | 0 |
| A. versicolor | 2019 | 1 | HC | 0.1201 | 1 |
| A. versicolor | 2019 | 1 | HC | 0.1573 | 2 |
| A. versicolor | 2019 | 1 | HC | 0.2792 | 3 |
| A. versicolor | 2019 | 1 | HC | 0 | 0 |
| A. versicolor | 2019 | 2 | HC | 0 | 0 |
| A. versicolor | 2019 | 2 | HC | 0.1574 | 2 |
| A. versicolor | 2019 | 2 | HC | 0.2147 | 3 |
| A. versicolor | 2019 | 2 | HC | 0.1658 | 2 |
| A. versicolor | 2019 | 1 | HS | 0 | 0 |
| A. versicolor | 2019 | 1 | HS | 0 | 0 |
| A. versicolor | 2019 | 1 | HS | 0 | 0 |
| A. versicolor | 2019 | 1 | HS | 0 | 0 |
| A. versicolor | 2019 | 2 | HS | 0 | 0 |
| A. versicolor | 2019 | 2 | HS | 0 | 0 |
| A. versicolor | 2019 | 2 | HS | 0 | 0 |
| A. versicolor | 2019 | 2 | HS | 0 | 0 |
| A. mekongensis | 2019 | 1 | O | 0.2842 | 4 |
| A. mekongensis | 2019 | 1 | O | 0 | 0 |
| A. mekongensis | 2019 | 1 | O | 0 | 0 |
| A. mekongensis | 2019 | 1 | OE | 0.2473 | 3 |
| A. mekongensis | 2019 | 1 | HC | 0.2507 | 3 |
| A. mekongensis | 2019 | 1 | HS | 0 | 0 |
| A. mekongensis | 2019 | 1 | C | 0 | 0 |
| A. mekongensis | 2019 | 2 | O | 0.2933 | 4 |
| A. mekongensis | 2019 | 2 | O | 0.2174 | 4 |
| A. mekongensis | 2019 | 2 | OE | 0 | 0 |
| A. mekongensis | 2019 | 2 | HC | 0 | 0 |
| A. mekongensis | 2019 | 2 | HS | 0 | 0 |
| A. mekongensis | 2019 | 2 | C | 0 | 0 |
| A. mekongensis | 2019 | 3 | O | 0 | 0 |
| A. mekongensis | 2019 | 3 | O | 0.1983 | 3 |
| A. mekongensis | 2019 | 3 | O | 0.219 | 4 |
| A. mekongensis | 2019 | 3 | O | 0.1624 | 0 |
| A. mekongensis | 2019 | 3 | O | 0.2155 | 3 |
| A. mekongensis | 2019 | 3 | OE | 0.156 | 2 |
| A. mekongensis | 2019 | 3 | HC | 0 | 0 |
| A. mekongensis | 2019 | 3 | HS | 0 | 0 |
| A. mekongensis | 2019 | 3 | C | 0 | 0 |
| A. mekongensis | 2019 | 4 | O | 0.2121 | 3 |
| A. mekongensis | 2019 | 4 | O | 0.1004 | 3 |
| A. mekongensis | 2019 | 4 | OE | 0.1047 | 1 |
| A. mekongensis | 2019 | 4 | OE | 0 | 0 |
| A. mekongensis | 2019 | 4 | HC | 0 | 0 |
| A. mekongensis | 2019 | 4 | HS | 0 | 0 |
| A. mekongensis | 2019 | 4 | C | 0 | 0 |
| A. mekongensis | 2019 | 5 | O | 0 | 0 |
| A. mekongensis | 2019 | 5 | O | 0.2844 | 4 |
| A. mekongensis | 2019 | 5 | O | 0 | 0 |
| A. mekongensis | 2019 | 5 | O | 0 | 0 |
| A. mekongensis | 2019 | 5 | OE | 0.121 | 1 |
| A. mekongensis | 2019 | 5 | OE | 0.2229 | 3 |
| A. mekongensis | 2019 | 5 | HC | 0 | 0 |
| A. mekongensis | 2019 | 5 | HC | 0 | 0 |
| A. mekongensis | 2019 | 5 | HS | 0 | 0 |
| A. mekongensis | 2019 | 5 | HS | 0 | 0 |
| A. mekongensis | 2019 | 5 | C | 0 | 0 |
| A. mekongensis | 2019 | 5 | C | 0 | 0 |
| A. mekongensis | 2020 | 1 | O | 0 | 0 |
| A. mekongensis | 2020 | 1 | O | 0 | 0 |
| A. mekongensis | 2020 | 1 | O | 0 | 0 |
| A. mekongensis | 2020 | 1 | O | 0 | 0 |
| A. mekongensis | 2020 | 1 | O | 0.2311 | 2 |
| A. mekongensis | 2020 | 1 | OE | 0 | 0 |
| A. mekongensis | 2020 | 1 | C | 0 | 0 |
| A. mekongensis | 2020 | 1 | C | 0 | 0 |
| A. mekongensis | 2020 | 1 | HC | 0.2849 | 3 |
| A. mekongensis | 2020 | 1 | HC | 0.0819 | 1 |
| A. mekongensis | 2020 | 1 | HS | 0 | 0 |
| A. mekongensis | 2020 | 1 | HS | 0 | 0 |
| A. mekongensis | 2020 | 2 | O | 0.0965 | 1 |
| A. mekongensis | 2020 | 2 | OE | 0 | 0 |
| A. mekongensis | 2020 | 2 | C | 0 | 0 |
| A. mekongensis | 2020 | 2 | HC | 0 | 0 |
| A. mekongensis | 2020 | 2 | HS | 0 | 0 |
| A. mekongensis | 2020 | 3 | O | 0 | 0 |
| A. mekongensis | 2020 | 3 | OE | 0 | 0 |
| A. mekongensis | 2020 | 3 | C | 0 | 0 |
| A. mekongensis | 2020 | 3 | C | 0 | 0 |
| A. mekongensis | 2020 | 3 | HC | 0 | 0 |
| A. mekongensis | 2020 | 3 | HS | 0 | 0 |
| A. mekongensis | 2020 | 4 | O | 0.3008 | 4 |
| A. mekongensis | 2020 | 4 | O | 0.2536 | 3 |
| A. mekongensis | 2020 | 4 | OE | 0.2454 | 3 |
| A. mekongensis | 2020 | 4 | OE | 0.2098 | 3 |
| A. mekongensis | 2020 | 4 | C | 0 | 0 |
| A. mekongensis | 2020 | 4 | C | 0 | 0 |
| A. mekongensis | 2020 | 4 | HC | 0 | 0 |
| A. mekongensis | 2020 | 4 | HC | 0.246 | 3 |
| A. mekongensis | 2020 | 4 | HS | 0 | 0 |
| A. mekongensis | 2020 | 4 | HS | 0 | 0 |
| A. mekongensis | 2020 | 5 | O | 0 | 0 |
| A. mekongensis | 2020 | 5 | O | 0 | 0 |
| A. mekongensis | 2020 | 5 | OE | 0.2818 | 4 |
| A. mekongensis | 2020 | 5 | OE | 0.2039 | 3 |
| A. mekongensis | 2020 | 5 | C | 0 | 0 |
| A. mekongensis | 2020 | 5 | C | 0 | 0 |
| A. mekongensis | 2020 | 5 | HC | 0.2468 | 3 |
| A. mekongensis | 2020 | 5 | HC | 0 | 0 |
| A. mekongensis | 2020 | 5 | HS | 0 | 0 |
| A. mekongensis | 2020 | 5 | HS | 0 | 0 |

**Table S3. Raw data from pollinator observations.**

| **Plant Species** | **Year** | **Flower ID** | **Taxa** | **Number of Visits** | **Hours Observed** | **Visits per hour** |
| --- | --- | --- | --- | --- | --- | --- |
| A. versicolor | 2019 | 1 | X. latipes | 0 | 12 | 0 |
| A. versicolor | 2019 | 2 | X. latipes | 0 | 12 | 0 |
| A. versicolor | 2019 | 3 | X. latipes | 0 | 10 | 0 |
| A. versicolor | 2019 | 4 | X. latipes | 11 | 22 | 0.5 |
| A. versicolor | 2019 | 5 | X. latipes | 3 | 22 | 0.136364 |
| A. versicolor | 2019 | 6 | X. latipes | 23 | 21 | 1.095238 |
| A. versicolor | 2019 | 7 | X. latipes | 23 | 21 | 1.095238 |
| A. versicolor | 2019 | 8 | X. latipes | 16 | 21 | 0.761905 |
| A. versicolor | 2019 | 9 | X. latipes | 35 | 21 | 1.666667 |
| A. versicolor | 2019 | 10 | X. latipes | 68 | 22 | 3.090909 |
| A. versicolor | 2019 | 11 | X. latipes | 60 | 22 | 2.727273 |
| A. versicolor | 2019 | 12 | X. latipes | 46 | 22 | 2.090909 |
| A. versicolor | 2019 | 13 | X. latipes | 66 | 22 | 3 |
| A. versicolor | 2019 | 14 | X. latipes | 54 | 22 | 2.454545 |
| A. versicolor | 2019 | 15 | X. latipes | 0 | 12 | 0 |
| A. versicolor | 2019 | 16 | X. latipes | 0 | 12 | 0 |
| A. versicolor | 2019 | 17 | X. latipes | 0 | 12 | 0 |
| A. versicolor | 2019 | 18 | X. latipes | 0 | 12 | 0 |
| A. versicolor | 2019 | 19 | X. latipes | 71 | 11 | 6.454545 |
| A. versicolor | 2019 | 20 | X. latipes | 37 | 11 | 3.363636 |
| A. versicolor | 2019 | 21 | X. latipes | 63 | 11 | 5.727273 |
| A. versicolor | 2019 | 22 | X. latipes | 57 | 22 | 2.590909 |
| A. versicolor | 2020 | 1 | X. latipes | 0 | 14 | 0 |
| A. versicolor | 2020 | 1 | X. aestuans | 0 | 14 | 0 |
| A. versicolor | 2020 | 2 | X. latipes | 0 | 14 | 0 |
| A. versicolor | 2020 | 2 | X. aestuans | 0 | 14 | 0 |
| A. versicolor | 2020 | 3 | X. latipes | 0 | 14 | 0 |
| A. versicolor | 2020 | 3 | X. aestuans | 0 | 14 | 0 |
| A. versicolor | 2020 | 4 | X. latipes | 0 | 14 | 0 |
| A. versicolor | 2020 | 4 | X. aestuans | 0 | 14 | 0 |
| A. versicolor | 2020 | 5 | X. latipes | 0 | 14 | 0 |
| A. versicolor | 2020 | 5 | X. aestuans | 0 | 14 | 0 |
| A. versicolor | 2020 | 6 | X. latipes | 0 | 13 | 0 |
| A. versicolor | 2020 | 6 | X. aestuans | 0 | 13 | 0 |
| A. versicolor | 2020 | 7 | X. latipes | 0 | 13 | 0 |
| A. versicolor | 2020 | 7 | X. aestuans | 0 | 13 | 0 |
| A. versicolor | 2020 | 8 | X. latipes | 0 | 13 | 0 |
| A. versicolor | 2020 | 8 | X. aestuans | 0 | 13 | 0 |
| A. versicolor | 2020 | 9 | X. latipes | 0 | 13 | 0 |
| A. versicolor | 2020 | 9 | X. aestuans | 0 | 13 | 0 |
| A. versicolor | 2020 | 10 | X. latipes | 0 | 20 | 0 |
| A. versicolor | 2020 | 10 | X. aestuans | 0 | 20 | 0 |
| A. versicolor | 2020 | 11 | X. latipes | 0 | 20 | 0 |
| A. versicolor | 2020 | 11 | X. aestuans | 0 | 20 | 0 |
| A. versicolor | 2020 | 12 | X. latipes | 6 | 17 | 0.352941 |
| A. versicolor | 2020 | 12 | X. aestuans | 1 | 17 | 0.058824 |
| A. versicolor | 2020 | 13 | X. latipes | 7 | 17 | 0.411765 |
| A. versicolor | 2020 | 13 | X. aestuans | 2 | 17 | 0.117647 |
| A. versicolor | 2020 | 14 | X. latipes | 3 | 17 | 0.176471 |
| A. versicolor | 2020 | 14 | X. aestuans | 1 | 17 | 0.058824 |
| A. versicolor | 2020 | 15 | X. latipes | 9 | 24 | 0.375 |
| A. versicolor | 2020 | 15 | X. aestuans | 0 | 24 | 0 |
| A. versicolor | 2020 | 16 | X. latipes | 16 | 24 | 0.666667 |
| A. versicolor | 2020 | 16 | X. aestuans | 1 | 24 | 0.041667 |
| A. versicolor | 2020 | 17 | X. latipes | 17 | 24 | 0.708333 |
| A. versicolor | 2020 | 17 | X. aestuans | 0 | 24 | 0 |
| A. versicolor | 2020 | 18 | X. latipes | 11 | 24 | 0.458333 |
| A. versicolor | 2020 | 18 | X. aestuans | 1 | 24 | 0.041667 |
| A. versicolor | 2020 | 19 | X. latipes | 14 | 17 | 0.823529 |
| A. versicolor | 2020 | 19 | X. aestuans | 1 | 17 | 0.058824 |
| A. versicolor | 2020 | 20 | X. latipes | 30 | 9 | 3.333333 |
| A. versicolor | 2020 | 20 | X. aestuans | 0 | 9 | 0 |
| A. versicolor | 2020 | 21 | X. latipes | 18 | 9 | 2 |
| A. versicolor | 2020 | 21 | X. aestuans | 0 | 9 | 0 |
| A. versicolor | 2020 | 22 | X. latipes | 4 | 9 | 0.444444 |
| A. versicolor | 2020 | 22 | X. aestuans | 0 | 9 | 0 |
| A. versicolor | 2020 | 23 | X. latipes | 5 | 9 | 0.555556 |
| A. versicolor | 2020 | 23 | X. aestuans | 0 | 9 | 0 |
| A. versicolor | 2020 | 24 | X. latipes | 1 | 9 | 0.111111 |
| A. versicolor | 2020 | 24 | X. aestuans | 0 | 9 | 0 |
| A. versicolor | 2020 | 25 | X. latipes | 6 | 5 | 1.2 |
| A. versicolor | 2020 | 25 | X. aestuans | 1 | 5 | 0.2 |
| A. versicolor | 2020 | 26 | X. latipes | 5 | 5 | 1 |
| A. versicolor | 2020 | 26 | X. aestuans | 0 | 5 | 0 |
| A. versicolor | 2020 | 27 | X. latipes | 22 | 11 | 2 |
| A. versicolor | 2020 | 27 | X. aestuans | 0 | 11 | 0 |
| A. versicolor | 2020 | 28 | X. latipes | 23 | 11 | 2.090909 |
| A. versicolor | 2020 | 28 | X. aestuans | 0 | 11 | 0 |
| A. versicolor | 2020 | 29 | X. latipes | 0 | 3 | 0 |
| A. versicolor | 2020 | 29 | X. aestuans | 0 | 3 | 0 |
| A. versicolor | 2020 | 30 | X. latipes | 6 | 21 | 0.285714 |
| A. versicolor | 2020 | 30 | X. aestuans | 0 | 21 | 0 |
| A. versicolor | 2020 | 31 | X. latipes | 4 | 21 | 0.190476 |
| A. versicolor | 2020 | 31 | X. aestuans | 0 | 21 | 0 |
| A. versicolor | 2020 | 32 | X. latipes | 5 | 21 | 0.238095 |
| A. versicolor | 2020 | 32 | X. aestuans | 0 | 21 | 0 |
| A. versicolor | 2020 | 33 | X. latipes | 6 | 21 | 0.285714 |
| A. versicolor | 2020 | 33 | X. aestuans | 0 | 21 | 0 |
| A. versicolor | 2020 | 34 | X. latipes | 1 | 21 | 0.047619 |
| A. versicolor | 2020 | 34 | X. aestuans | 0 | 21 | 0 |
| A. versicolor | 2020 | 35 | X. latipes | 6 | 21 | 0.285714 |
| A. versicolor | 2020 | 35 | X. aestuans | 0 | 21 | 0 |
| A. versicolor | 2020 | 36 | X. latipes | 0 | 14 | 0 |
| A. versicolor | 2020 | 36 | X. aestuans | 0 | 14 | 0 |
| A. versicolor | 2020 | 37 | X. latipes | 0 | 14 | 0 |
| A. versicolor | 2020 | 37 | X. aestuans | 0 | 14 | 0 |
| A. versicolor | 2020 | 38 | X. latipes | 0 | 14 | 0 |
| A. versicolor | 2020 | 38 | X. aestuans | 0 | 14 | 0 |
| A. versicolor | 2020 | 39 | X. latipes | 0 | 14 | 0 |
| A. versicolor | 2020 | 39 | X. aestuans | 0 | 14 | 0 |
| A. versicolor | 2020 | 40 | X. latipes | 0 | 14 | 0 |
| A. versicolor | 2020 | 40 | X. aestuans | 0 | 14 | 0 |
| A. versicolor | 2020 | 41 | X. latipes | 1 | 11 | 0.090909 |
| A. versicolor | 2020 | 41 | X. aestuans | 1 | 11 | 0.090909 |
| A. versicolor | 2020 | 42 | X. latipes | 1 | 11 | 0.090909 |
| A. versicolor | 2020 | 42 | X. aestuans | 0 | 11 | 0 |
| A. versicolor | 2020 | 43 | X. latipes | 0 | 9 | 0 |
| A. versicolor | 2020 | 43 | X. aestuans | 15 | 9 | 1.666667 |
| A. versicolor | 2020 | 44 | X. latipes | 0 | 9 | 0 |
| A. versicolor | 2020 | 44 | X. aestuans | 14 | 9 | 1.555556 |
| A. versicolor | 2020 | 45 | X. latipes | 0 | 9 | 0 |
| A. versicolor | 2020 | 45 | X. aestuans | 9 | 9 | 1 |

| **Plant Species** | **Year** | **Plant ID** | **Taxa** | **Number of Visits** | **Hours Observed** | **Visits per hour** |
| --- | --- | --- | --- | --- | --- | --- |
| A. mekongensis | 2019 | 1 | X. latipes | 2 | 18 | 0.111111 |
| A. mekongensis | 2019 | 1 | X. aestuans | 0 | 18 | 0 |
| A. mekongensis | 2019 | 2 | X. latipes | 46 | 45 | 1.022222 |
| A. mekongensis | 2019 | 2 | X. aestuans | 26 | 45 | 0.577778 |
| A. mekongensis | 2019 | 3 | X. latipes | 21 | 62 | 0.33871 |
| A. mekongensis | 2019 | 3 | X. aestuans | 14 | 62 | 0.225806 |
| A. mekongensis | 2019 | 4 | X. latipes | 9 | 28 | 0.321429 |
| A. mekongensis | 2019 | 4 | X. aestuans | 2 | 28 | 0.071429 |
| A. mekongensis | 2019 | 5 | X. latipes | 3 | 21 | 0.142857 |
| A. mekongensis | 2019 | 5 | X. aestuans | 18 | 21 | 0.857143 |
| A. mekongensis | 2020 | 1 | X. latipes | 17 | 20 | 0.85 |
| A. mekongensis | 2020 | 1 | X. aestuans | 12 | 20 | 0.6 |
| A. mekongensis | 2020 | 1 | unkn.1 | 0 | 20 | 0 |
| A. mekongensis | 2020 | 1 | unkn.2 | 0 | 20 | 0 |
| A. mekongensis | 2020 | 2 | X. latipes | 37 | 35 | 1.057143 |
| A. mekongensis | 2020 | 2 | X. aestuans | 48 | 35 | 1.371429 |
| A. mekongensis | 2020 | 2 | unkn.1 | 0 | 35 | 0 |
| A. mekongensis | 2020 | 2 | unkn.2 | 0 | 35 | 0 |
| A. mekongensis | 2020 | 3 | X. latipes | 0 | 14 | 0 |
| A. mekongensis | 2020 | 3 | X. aestuans | 0 | 14 | 0 |
| A. mekongensis | 2020 | 3 | unkn.1 | 0 | 14 | 0 |
| A. mekongensis | 2020 | 3 | unkn.2 | 0 | 14 | 0 |
| A. mekongensis | 2020 | 4 | X. latipes | 1 | 37 | 0.027027 |
| A. mekongensis | 2020 | 4 | X. aestuans | 19 | 37 | 0.513514 |
| A. mekongensis | 2020 | 4 | unkn.1 | 5 | 37 | 0.135135 |
| A. mekongensis | 2020 | 4 | unkn.2 | 2 | 37 | 0.054054 |
| A. mekongensis | 2020 | 5 | X. latipes | 0 | 29 | 0 |
| A. mekongensis | 2020 | 5 | X. aestuans | 0 | 29 | 0 |
| A. mekongensis | 2020 | 5 | unkn.1 | 0 | 29 | 0 |
| A. mekongensis | 2020 | 5 | unkn.2 | 0 | 29 | 0 |
